# Comparison of T2 and FLAIR imaging for target delineation in high grade gliomas

**DOI:** 10.1186/1748-717X-5-5

**Published:** 2010-01-28

**Authors:** Bronwyn Stall, Leor Zach, Holly Ning, John Ondos, Barbara Arora, Uma Shankavaram, Robert W Miller, Deborah Citrin, Kevin Camphausen

**Affiliations:** 1 Radiation Oncology Branch, National Cancer Institute, 10 Center Drive, Building 10, CRC, Rm B2-3561, Bethesda, MD, 20892 USA

## Abstract

**Background:**

FLAIR and T2 weighted MRIs are used based on institutional preference to delineate high grade gliomas and surrounding edema for radiation treatment planning. Although these sequences have inherent physical differences there is limited data on the clinical and dosimetric impact of using either or both sequences.

**Methods:**

40 patients with high grade gliomas consecutively treated between 2002 and 2008 of which 32 had pretreatment MRIs with T1, T2 and FLAIR available for review were selected for this study. These MRIs were fused with the treatment planning CT. Normal structures, clinical tumor volume (CTV) and planning tumor volume (PTV) were then defined on the T2 and FLAIR sequences. A Venn diagram analysis was performed for each pair of tumor volumes as well as a fractional component analysis to assess the contribution of each sequence to the union volume. For each patient the tumor volumes were compared in terms of total volume in cubic centimeters as well as anatomic location using a discordance index. The overlap of the tumor volumes with critical structures was calculated as a measure of predicted toxicity. For patients with MRI documented failures, the tumor volumes obtained using the different sequences were compared with the recurrent gross tumor volume (rGTV).

**Results:**

The FLAIR CTVs and PTVs were significantly larger than the T2 CTVs and PTVs (p < 0.0001 and p = 0.0001 respectively). Based on the discordance index, the abnormality identified using the different sequences also differed in location. Fractional component analysis showed that the intersection of the tumor volumes as defined on both T2 and FLAIR defined the majority of the union volume contributing 63.6% to the CTV union and 82.1% to the PTV union. T2 alone uniquely identified 12.9% and 5.2% of the CTV and PTV unions respectively while FLAIR alone uniquely identified 25.7% and 12% of the CTV and PTV unions respectively. There was no difference in predicted toxicity to normal structures using T2 or FLAIR. At the time of analysis, 26 failures had occurred of which 19 patients had MRIs documenting the recurrence. The rGTV correlated best with the FLAIR CTV but the percentage overlap was not significantly different from that with T2. There was no statistical difference in the percentage overlap with the rGTV and the PTVs generated using either T2 or FLAIR.

**Conclusions:**

Although both T2 and FLAIR MRI sequences are used to define high grade glial neoplasm and surrounding edema, our results show that the volumes generated using these techniques are different and not interchangeable. These differences have bearing on the use of intensity modulated radiation therapy (IMRT) and highly conformal treatment as well as on future clinical trials where the bias of using one technique over the other may influence the study outcome.

## Introduction

According to the Central Brain Tumor Registry of the United States, more than 20,000 malignant brain tumors are diagnosed each year. Glioblastoma Multiforme (GBM) accounts for 70% of new adult cases of malignant brain tumors. While this represents only 1.4% of all primary malignant tumors in the US, the poor 5 year survival rate of less than 4% has commanded extensive clinical research [1].

Standard primary therapy for high grade gliomas includes maximal safe resection followed by adjuvant radiation and chemotherapy. As imaging techniques have advanced over the past several decades, targeting for radiotherapy has evolved to include new modalities in treatment planning. The use of these complementary imaging modalities in treatment planning and assessment may allow more accurate targeting of tumor, improved sparing of normal tissues, and early assessment of disease response to therapy.

The foundation of radiation treatment planning for GBM is based on landmark studies demonstrating predilection for central recurrence and data correlating pathologic findings with imaging abnormalities [2-5]. Contemporary radiation therapy planning for high grade gliomas involves identifying tumor volumes on various MRI sequences. Institutional preference generally dictates whether T2 or FLAIR is used to define tumor volumes and associated edema. Because there is limited data comparing the dosimetric and clinical impact of using these imaging sequences for radiotherapy planning, we aimed to evaluate the differences in terms of treatment volumes, changes in dose distribution to critical structures, and effects on clinical outcome.

## Materials and methods

### Treatment Planning

We used treatment planning images of all adult patients with high grade gliomas treated between 2002 and 2008 at the National Cancer Institute in whom a complete pretreatment MRI with contrast-enhanced T1, T2 and FLAIR sequences was currently available for review. Demographic factors were reviewed for prognostic data including, age, functional status, extent of resection prior to treatment and a recursive partitioning analysis (RPA) score was calculated for all patients based on these factors [6,7].

All patients were simulated for radiation treatment planning with immobilization via a custom thermoplastic face mask. CT imaging of the head and upper neck was performed using a Philips Large Bore CT scanner and images were transferred to a Varian Eclipse planning system (version 6.5). A 3D volume was created for each patient from the treatment planning CT. All MRI sequences were fused to this 3D volume. Match points were used to align analogous anatomic landmarks on the CT and MRI. 3D translations and rotations were then performed and visually verified in axial, sagittal and coronal views. Each fusion was approved by the physicist and treating physician.

**Figure 1:**
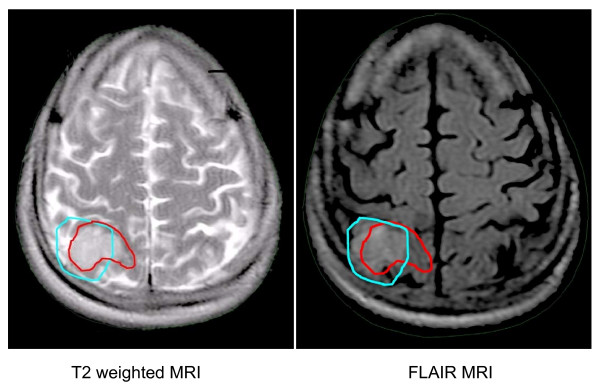
**Overlay of tumor volumes as contoured on T2 and FLAIR sequences**. The T2 abnormality is contoured in red and the FLAIR abnormality is contoured in cyan.

Once a satisfactory fusion was achieved, normal structures and tumor volumes were contoured on the T2 and FLAIR sequences without comparison to the alternative sequence (Figure 1). The clinical tumor volume (CTV) consisted of the enhancing lesion and surrounding edema. A 2 cm volumetric expansion of the CTV was delineated as the planning tumor volume (PTV). Using the "calculate volume" function, the target volumes in cubic centimeters (cc) for all patients were recorded. For each patient the difference between the target volumes were tabulated and a mean percent difference was then calculated for the target volumes. The union (the area belonging to one or both of the defined volumes) and intersection (the area belonging to both defined volumes) of the CTVs and PTVs were determined using a Boolean function. These values were then used to calculate the fractional component contributed by the imaging techniques as previously described by Haken and colleagues [8,9].

As a means of incorporating the data from each of the sequences, a combined PTV was created from the union of the T2 and FLAIR CTVs with a standard 2 cm volumetric expansion. The percent difference and absolute difference between the combined PTV and the T2 and FLAIR PTV was calculated.

Next we investigated the potential consequences in respect to normal tissue exposure using the PTVs generated with different MRI sequences. Because of the retrospective design of this study, we evaluated the overlap of the PTVs with normal structures as a surrogate for toxicity. It is probable that most clinicians would trim target volumes to avoid overdosing normal tissue; however, this metric provides data on the likelihood of normal tissue coverage by the PTV. The brainstem and chiasm were selected as at risk for critical exposure based on historical tissue tolerance data [10]. Inclusion of these organs within the PTV was defined as a high risk of a critical exposure and was determined by using the Boolean operator function to find the intersection in cubic centimeters of the T2 and FLAIR PTVs with the brainstem and chiasm. The number of patients with critical structure overlaps as well as the percentage overlaps with the PTV as defined by T2 and FLAIR was recorded.

For comparison purposes, the tumor volumes were evaluated in pairs (e.g. CTV as delineated on T2 and FLAIR). Each pair of target volumes was compared on the basis of total volume as well as anatomic location. The volume in cubic centimeters was determined using the "calculate volume" function on the planning software. To assess the differences in location, a discordance index was calculated. This was defined as the union of the two volumes minus the ratio of the intersection to the union: (A U B) - (A n B)/(A U B).

Finally, for patients with MRI documented brain failures we looked at the recurrence patterns both in terms of their relationship to the tumor volumes delineated using the different MRI sequences as well as to the delivered dose. Specifically, the T1 sequence was fused to the original treatment planning CT and the recurrence volume was delineated as the recurrent gross tumor volume (rGTV). The overlap of the rGTV with each of the planning volumes was calculated using the intersection Boolean operator function. The centrality of failure was determined by overlaying the delivered dose distribution on the planning CT. If the rGTV was encompassed by the 95% isodose line the failure was scored as central [11].

### Statistics

Because of the large range in tumor volumes, the CTV and PTV for each MRI sequence were normalized to their respective union volumes. This allowed for comparison using a two tailed paired student t-test.

## Results

### Patient Characteristics

Over the study period, 32 adult patients with high grade gliomas were treated with definitive radiotherapy at the NCI that had the required pretreatment contrast-enhanced MR with T1, T2 and FLAIR sequences. All pathology was reviewed at the NCI prior to treatment, with the majority of patients documented to have world health organization (WHO) grade IV gliomas (26/32). The remaining 6 patients had anaplastic astrocytomas. The remaining clinical demographics are summarized in Table 1.

**Table 1:** Summary of Patient Characteristics

Characteristic	
Age (median)	54 (30-73)
	
Sex	
Women	12
Men	20
	
WHO Grade	
III	7
IV	26
	
RPA Class	
I	3
II	0
III	7
IV	12
V	8
VI	2
	
Concurrent therapy	
None	9
TMZ	15
TMZ/valproic acid	8

*Abbreviations*: WHO-World Health

Organization, RPA-Recursive Partitioning

Analysis, TMZ-temozolamide

Nine patients treated prior to the landmark study published by Stupp *et al *in 2005 received radiation treatment alone; following this publication, patients received concurrent and adjuvant temozolomide [12]. Eight patients were enrolled on a phase II study of the histone deacetylase inhibitor valproic acid in combination with standard temozolomide therapy. The acute toxicity results have since been presented [13]. In total, twenty-three patients received temozolomide. All patients were treated with 3D conformal plans to a median dose of 60 Gy.

### T2 and FLAIR volumes

There was a large range in the size of CTVs and PTVs across the patient cohort. The mean T2 and FLAIR CTVs were 98.99 cc (range 1.51-383.5 cc) and 113.76 cc (range 2.77-546 cc), respectively. The mean T2 and FLAIR PTVs were 486.11 (91.81-1233.82) and 523.38 (101.89-1458.51). The mean percent difference between the CTV volumes from T2 images was 21% and the mean percent difference between the PTV volumes from the same data sets was 9%. To account for the large range in values, each volume was normalized to the union and compared using a two tailed paired student t-test. The FLAIR volumes were significantly larger than those obtained with T2 (p < 0.0001 for CTV and p = 0.0001 for PTV).

**Figure 2:**
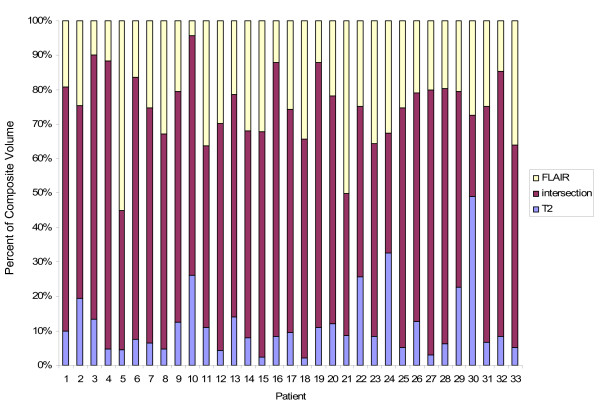
**Fractional component of the CTV Union**. The contribution from T2 alone is in blue, the contribution from FLAIR alone is in yellow and the intersection is in maroon.

**Figure 3:**
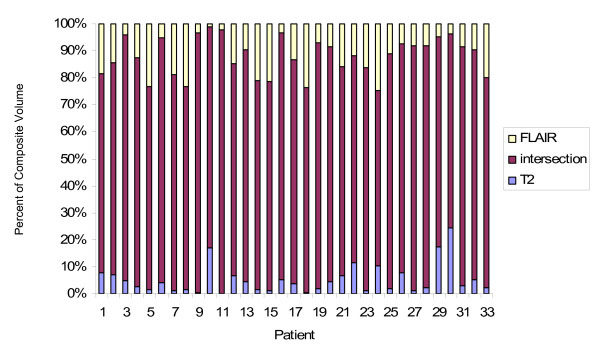
**Fractional component of the PTV Union**. The contribution from T2 alone is in blue, the contribution from FLAIR alone is in yellow and the intersection is in maroon.

The average overlap (intersection) of the T2 and FLAIR CTVs was 83.84 cc and the average union was 126.34 cc. The average intersection of the T2 and FLAIR PTVs was 452.32 and the average union was 553.69 cc. There was a large range of discordance between the CTVs and PTVs. The CTV discordance ranged from 0.083 - 0.65 with an average of 0.359 (std dev 0.13). With a volumetric expansion to create the PTV, the average discordance decreased to 0.20 with a range of 0.079 -1 (std dev 0.15). The average fractional component of the CTV Union was 12.9% for T2 and 25.7% for FLAIR, while the overlap contributed the majority (63.6%) as shown in figure 2. For example, the composite CTV for patient 1 was comprised of 10% T2 only, 19% FLAIR only and 71% by the intersection of the T2 and FLAIR volumes. Similarly, the largest component of the PTV Union was the overlap (82.1%) while T2 and FLAIR contributed 5.2% and 12%, respectively (figure 3). Thus although the largest component of the union volumes was the intersection, each sequence contributed unique information

### Combined Planning Tumor Volumes

The average combined PTV, created from the union of the T2 and FLAIR CTVs with a 2 cm volumetric expansion was 514.20 cc (104.28 - 178.96 cc). The mean volume difference between the combined PTV and the T2 and FLAIR PTVs was 46.23 cc and 18.67 cc. Using the combined PTV would result in an 11.7% variation from the PTV as defined by T2 and 4.16% as defined by FLAIR.

### Normal Structure Toxicity

Using FLAIR to define the PTV, 19 patients had overlap with the brainstem as compared to 23 using T2. Numerically, the average percent overlap with FLAIR was higher; however this was not statistically significant. The overlap was 32% and 26%, respectively, for FLAIR and T2 (p = 0.81). Volumetrically, the average overlap with the brain stem was 5.27 cc using FLAIR and 4.88 cc using T2. Similarly, slightly more patients had overlap with the chiasm on FLAIR, 11 versus 9. This percent overlap was not statistically significant, 26% vs 34% (p = 0.18) with the T2 overlap being greater. The average overlap with the brainstem was 0.091 cc using FLAIR and 0.085 cc using T2. Based on this surrogate analysis, using FLAIR rather than T2 to delineate tumor volumes could inherently increase toxicity.

### Failure Data

**Figure 4:**
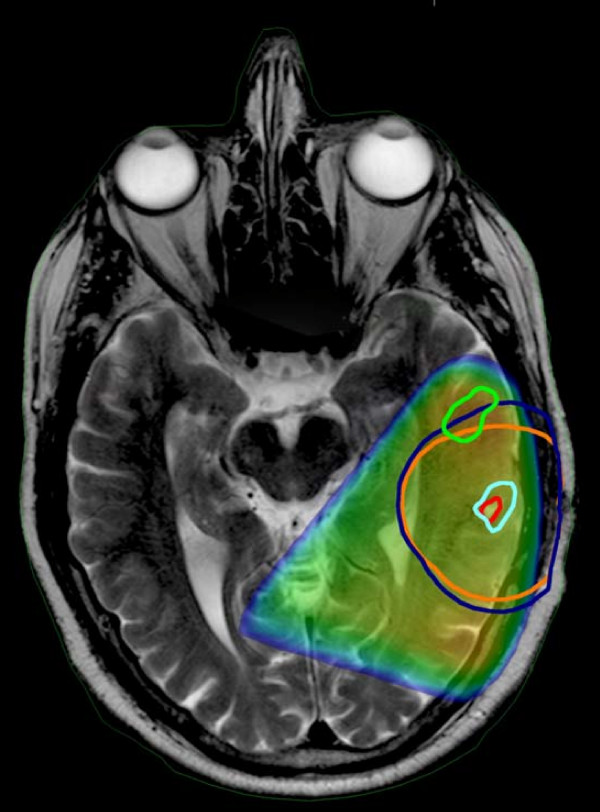
**Planning T2 MRI fused with FLAIR images from same date and T1 MRI obtained at time of failure**. The failure volume (rGTV) is contoured in light green. The T2 and FLAIR CTVs are outlined in red and cyan respectively. The T2 and FLAIR PTVs are outlined in orange and dark blue respectively. The FLAIR PTV encompasses a greater portion of the failure volume than T2 PTV. Overlay of the 95% dose color wash shows that the failure is central.

At the time of analysis, 26 failures had occurred of which 19 patients had MR images available from the time of failure. Two patients are alive and 4 are lost to follow-up. As expected based on literature reporting recurrence patterns, all failures were central as defined by coverage by the 95% isodose line [2,5,14]. Fourteen of the 19 failures were entirely encompassed by both the T2 and FLAIR PTV. The remaining failures were partially encompassed with three failures corresponding better with FLAIR and two failures with T2 images. An example of this analysis is shown in figure 4. Numerically, the percent overlap of the failure GTV was greater with the FLAIR CTV than with the T2 CTV, however this was not statistically significant p = 0.1. This was also true of the PTV overlap with failure GTV, p = 0.6.

As suggested by the fractional component analysis, both sequences provide unique information about the diseased tissue and therefore provide different but valid information regarding the site of future recurrences.

## Discussion

Imaging of malignant brain tumors has played an important role in radiation treatment planning. Within years of the landmark discovery by Roentgen [15], the use of radiographs to diagnose cerebral tumors became routine [16]. However, the relative limited resolution and accuracy of plain radiographs and other early imaging modalities such as ventriculography and angiography supported the use of whole brain treatment [17-22]. It was not until the early 1970's that partial brain treatment became a viable option with the introduction of CT which heralded a dramatic change in the diagnostic evaluation and treatment principles of gliomas. The correlation of CT imaging and histological data in conjunction with clinical data demonstrating 80% of local recurrence arising within 2 cm of the original tumor as defined by CT, paved the foundation for successful partial brain treatment [2,3,5,14,23-28].

Nearly concurrent with introduction of CT imaging, MRI was developed and quickly became an important tool for radiation treatment planning. Biopsy evaluation identified tumor cells in the area of MRI T2 abnormality outside the contrast enhancing CT abnormality [4] and was subsequently incorporated into the target volume for radiation treatment planning [8,9]. While T2 MRI improved delineation of the extent of microscopic disease, several limitations became apparent. Specifically, T2 weighting causes CSF to be brighter than the brain and can be degraded by volume averaging and fluid motion artifacts secondary to normal cardiopulmonary cycles. These disadvantages led to the development of the FLAIR sequence [29]. By nullifying the CSF signal and decreasing the contrast between gray and white matter, the conspicuity of lesions in the periventricular and peripheral subcortical areas was improved [30]. Current Radiation Therapy Oncology Group protocols advise using CT and either FLAIR or T2 images to identify tumor volumes [31]. However, the differences in T2 and FLAIR MRI sequences to delineate clinically significant tumor burden have not been clearly defined in radiation treatment planning for high grade gliomas.

The results of this study demonstrate both a qualitative and quantitative difference between the tumor target volumes as defined by T2 and FLAIR. The volumes of both the CTV and PTV delineated using FLAIR were significantly larger than those obtained using T2. Despite this increase in size, there was not a significant difference in the overlap with critical structures suggesting that incorporating the FLAIR abnormality does not necessarily increase toxicity. The discordance index between these techniques was substantial, indicating geographic differences in the visualized abnormality. The majority of the target composite volumes were seen on both the T2 and FLAIR images. However, both sequences contributed unique and equally valid data to the composite volume. With regards to the patterns of failure, most lesions were encompassed by both T2 and FLAIR but several patients' lesions only correlated with one sequence. It is known from the underlying physics that the FLAIR technique nullifies CSF, but it is unclear if other factors may account for the differences between FLAIR and T2.

Other investigators have evaluated the utility of incorporating additional imaging techniques into glioma planning but to our knowledge there is no data regarding the differences using T2 versus FLAIR to delineate high grade gliomas for radiation treatment. Functional imaging such as IMP-SPECT, MRSI, and PET have shown promise in guiding treatment planning as well as predicting response. Similar to our results, studies of these techniques have shown extension beyond the T2 abnormality suggesting that traditional targeting may be inadequate [32-34]. However, the incorporation of these novel advances may be limited by availability and cost while FLAIR is readily accessible.

We recognize several limitations in our study. This a retrospective review of a small cohort. As such, the time between diagnostic MRI and simulation CT as well as the use or dosing of steroids was not controlled and may have influenced our results. In assessing differences between the FLAIR and T2 volumes we did not correct for image registration errors. However, based on our comparison of T2 and FLAIR imaging for radiation treatment planning, both techniques are important and not interchangeable. Each technique can help distinguish normal parenchyma from edema and abnormal tissue. FLAIR is inherently more complex as it includes some T1 weighted effects. Our results do not show one technique to be superior but suggest such differences should not be ignored in high grade treatment conformal or IMRT planning, especially within a clinical trial where the results may be biased by the preference of one sequence over the other.

## Competing interests

The authors declare that they have no competing interests.

## Authors' contributions

BS participated in the image fusion, performed the contouring, participated in the data analysis and wrote the manuscript. LZ participated in contouring and helped revise the draft manuscript. HN checked image fusion and participated in treatment planning. JO carried out MRI fusions and participated in treatment planning. BA participated in treatment planning and checked image fusion. US participated in the statistical analysis of the results. RWM participated in treatment planning. DC participated in study design, data analysis and helped revise the draft manuscript. KC conceived of the study, and participated in its design and coordination and helped to draft the manuscript. All authors read and approved the final manuscript.
